# New avenues for systematically inferring cell-cell communication: through single-cell transcriptomics data

**DOI:** 10.1007/s13238-020-00727-5

**Published:** 2020-05-21

**Authors:** Xin Shao, Xiaoyan Lu, Jie Liao, Huajun Chen, Xiaohui Fan

**Affiliations:** 1 College of Pharmaceutical Sciences, Zhejiang University, Hangzhou, 310058 China; 2 College of Computer Science and Technology, Zhejiang University, Hangzhou, 310027 China; 3 The First Affiliated Hospital, School of Medicine, Zhejiang University, Hangzhou, 310003 China; 4 The Save Sight Institute, Faculty of Medicine and Health, The University of Sydney, Sydney, NSW 2000 Australia

**Keywords:** cell-cell communication, single-cell RNA sequencing, physical contact-dependent communication, chemical signal-dependent communication, ligand-receptor interaction, network biology

## Abstract

For multicellular organisms, cell-cell communication is essential to numerous biological processes. Drawing upon the latest development of single-cell RNA-sequencing (scRNA-seq), high-resolution transcriptomic data have deepened our understanding of cellular phenotype heterogeneity and composition of complex tissues, which enables systematic cell-cell communication studies at a single-cell level. We first summarize a common workflow of cell-cell communication study using scRNA-seq data, which often includes data preparation, construction of communication networks, and result validation. Two common strategies taken to uncover cell-cell communications are reviewed, e.g., physically vicinal structure-based and ligand-receptor interaction-based one. To conclude, challenges and current applications of cell-cell communication studies at a single-cell resolution are discussed in details and future perspectives are proposed.

## Introduction

Cell-cell communication, also known as cell-cell interaction, is an essential feature of multicellular organisms (Singer 1992). The dynamic communicating network formed through communication and cooperation between cells plays crucial roles in numerous biological processes (Petersen et al., 1999; Kirouac et al., 2009; Wang et al., 2013). Macrophages in the bone marrow are known to directly interact with erythroblasts in erythroblastic islands to facilitate their maturation (Ramos et al., 2013), while cancer-associated fibroblasts (CAFs) collaborate with tumor-associated macrophages (TAMs) in the tumor microenvironment to promote tumor progression (Kumar et al., 2017). Increasing evidence has demonstrated extensive cellular heterogeneity within a group cells and the existence of previously unknown cell types (Cheow et al., 2016). Therefore, investigation of cell-cell communication at a single-cell resolution within complex tissues remains a challenge.

Fortunately, recent advances in single-cell RNA sequencing (scRNA-seq) have enabled the simultaneous classification of thousands of cells in a single assay based on transcriptome profiling (Macosko et al., 2015; Klein et al., 2015), which result in the characterization of several novel or rare cell-types that have been limitedly reported (Grun et al., 2015). For example, megakaryocyte-erythroid progenitor cells from the bone marrow (Nestorowa et al., 2016), *Lgr5*-positive stem cells from the intestine (Gao et al., 2018), and type I spiral ganglion neurons from the mouse brain (Shrestha et al., 2018). These advances have shed light on the understanding of cellular heterogeneity and provided the means to investigate unknown cell-cell commutations through single-cell transcriptomics data systematically. Recently some progresses have been made to identify intercellular communication using scRNA-seq methodologies. Here we first describe general workflow of scRNA-seq procedures for cell-cell communication studies, including data preparation, construction of cell-cell communicating networks, computational analysis and validation of results. Two common strategies, e.g., physically vicinal structure-based and ligand-receptor interaction-based strategies according to physical contact-dependent and chemical signal-dependent communications, are reviewed in details. Finally, we present the current applications and challenges by investigating cell-cell communication from single-cell level as well as the future perspectives of this field.

## General workflow

**Figure 1:**
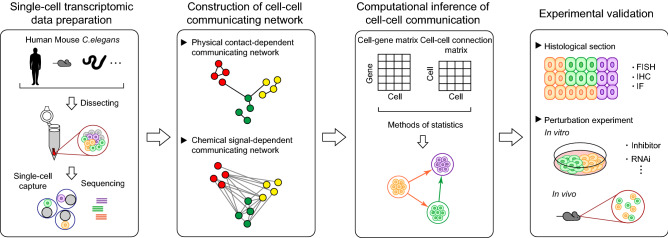
**General procedures of cell-cell communication studies using scRNA-seq techniques**. Human, mouse, or *C*. *elegans* samples were first dissected followed by scRNA-seq analysis to obtain the single-cell transcriptomic data, prepared for construction of cell-cell communicating network. Physical contact-dependent and chemical signal-dependent communicating networks constitute the two kinds of the cell-cell communicating network. In combination with statistical analysis, a cell-gene matrix generated from scRNA-seq protocols and a cell-cell connection matrix generated from the cell-cell communicating network are integrated to investigate cell-cell communications at a single-cell resolution. For experimental validation of inferred cell-cell communications, histological sections are evaluated to verify the physical contact-dependent cell-cell communications based on physically vicinal structure of cells, while perturbation experiments under inhibiting conditions are applied to verify the chemical signal-dependent cell-cell communications based on ligand-receptor interactions

For most cell-cell communication studies with scRNA-seq techniques, the common workflow mainly starts from data preparation followed by the construction of cell-cell communicating networks and the computationally inferring cell-cell communications. The last step is the validation of inferred cell-cell communications (Fig. 1). Briefly, during the data acquisition, the single-cell transcriptomic data of the specimen are collected through various scRNA-seq platforms, including the widely-used 10x Genomics (Zheng et al., 2017), as well as CEL-seq (Hashimshony et al., 2012), Smart-seq2 (Picelli et al., 2013), MARS-seq (Jaitin et al., 2014), etc. (Table 1). Single-cell identity needs to be annotated for further analysis. The common practice is to identify cell types with the known cell markers based on the pre-computed cell clusters such as scCATCH (Shao et al., 2020), or to mark cells by comparing the similarity of single-cell expression profiles with reference database using SingleR Aran et al. (2019) and scMap (Kiselev et al., 2018), prepared for the subsequent the construction of cell-based or cell-type-based cell-cell communicating network.

**Table 1:** Cell-cell communication studies using scRNA-seq techniques

Study	Tissue	Condition	scRNA-seq platform	Strategy
Boisset et al. (2018)	Bone marrow, small intestine	Healthy	CEL-Seq	(a)
Szczerba et al. (2019)	Blood	Breast cancer	Microfluidics; sgRNA sequencing	(a)
Martin et al. (2019)	Ileal tissue	iCD disease	10x Genomics	(b)
Kumar et al. (2018)	NA	Tumor	10x Genomics	(b)
Vento et al. (2018)	Fetal placenta	Healthy	10x Genomics; Smart-seq2	(b)
Hu et al. (2019)	Fetal NR and RPE	Healthy	STRT protocol	(b)
Fernandez et al. (2019)	Plaque and blood	Atherosclerosis	10x Genomics	(b)
Skelly et al. (2018)	Heart	Healthy	10x Genomics	(b)
Wang et al. (2019)	Blood	Healthy	Fluidigm C1; FACS	(b)
Camp et al. (2017)	liver bud organoids	Healthy	Fluidigm C1	(b)
Cohen et al. (2018)	Lung	Development	MARS-seq	(b)
Xiong et al. (2019)	Liver	NASH; Healthy	10x Genomics	(b)
Zhang et al. (2018)	Oocytes and GCs	Healthy	mRNA-Seq	(b)
Zepp et al. (2017)	Lung	Healthy	10x Genomics	(b)
Duan et al. (2018)	Brain	Inflammation	10x Genomics	(b)
Li et al. (2017)	Fetal gonad	Healthy	Modified Smart-seq2	(b)
Rajbhandari et al. (2019)	Adipocytes	Obesity	10x Genomics	(b)

NA, not available

(a) Physically vicinal structure-based strategy; (b) Ligand-receptor interaction-based strategy

In the cell-cell communicating network, nodes usually represent cells or cell types and the edges denote the physical connections (spatial neighbors) or the chemical connections (ligand-receptor interactions) between two cells or cell types. For the study by Boisset et al. (2018) and Szczerba et al. (2019), a physically connected cell-cell communicating network was constructed based on the physically vicinal structure of cells (Table 2). However, more efforts focus on the ligand-receptor-interaction-based communication network construction and analysis between cell types or cells, which present in the studies by Martin et al. (2019), Kumar et al. (2018), Vento-Tormo et al. (2018), Hu et al. (2019), Fernandez et al. (2019), Skelly et al. (2018), Wang et al. (2019) and Camp et al. (2017). Cohen et al. (2018) analyzed the correlation of ligand and receptor gene expression between pair-wise meta-cells and built the cell-type-specific ligand-receptor correlation network to define the cell-cell communications, while (Xiong et al., 2019) constructed the heterogeneous cell-type-ligand-receptor communicating network by filtering highly expressed ligands and receptor for each cell types (Table 2). Statistical analysis on the cell-cell communicating network identifies significant cell-cell communications between cell-types. In the work by Boisset et al. (2018), a 10,000 times permutation test on the vertices of physically connected cell-cell communicating network was performed using random sampling of cells, which leads to a distribution for each type of interaction in the network. Significantly enriched and depleted interactions (*P* < 0.05) are found by the comparison with the experimental number of interactions. For ligand-receptor-interaction-based communication network, significantly enriched ligand-receptor pairs between cell types or cells are also defined by statistical methods such as the widely-used permutation test or Welch’s t-test, Wilcoxon rank-sum test and the probability model as shown in Table 2. Then the frequency statistics of those significantly enriched pairs is computed to infer potential cell-cell communications with the most ligand-receptor pairs. What’s more, some scRNA-seq-based studies on cell-cell communication, e.g., Zhang et al. (2018), Zepp et al. (2017), Duan et al. (2018), Dong et al. (Li et al., 2017) and Rajbhandari et al. (2019), majorly rely on the prior knowledge to define potential cell-cell communication, wherein the mechanism underlying these communications needs to be further elucidated at a single-cell resolution.

**Table 2:** Cell-cell communicating networks and computational analysis

Study	Networks	Computational analysis
Boisset et al. (2018)		Permutation test of randomly sampling cells and repeat 10,000 times to obtain a distribution for each type of interaction and compare the experimental number of interactions to define the significantly enriched and depleted interaction (*P* < 0.05)
Szczerba et al. (2019)		Frequency statistics of WBCs in all CTC-WBC clusters
Martin et al. (2019)		Frequency statistics of significantly enriched ligand-receptor pairs by comparing intensity scores of the pairs (product of normalized ligand and receptor gene expression) between cell types in patients with or without the GIMATS module using permutation test and Benjamini-Hochberg adjusted *P* < 0.01
Kumar et al. (2018)		Frequency statistics of significantly present ligand-receptor pairs by performing one-sided Wilcoxon rank-sum test (Benjamini-Hochberg false discovery rate < 0.33) on the interaction score (product of average ligand and receptor gene expression) between cell types
Vento-Tormo et al. (2018)		Frequency statistics of significantly enriched ligand-receptor pairs by comparing the mean expression of ligand and receptor between cell types with the simulated distribution from randomly permuting the cluster labels of all cells 1,000 times (*P* < 0.05) (CellPhoneDB)
Hu et al. (2019)		CellPhoneDB as described above
Fernandez et al. (2019)		Interaction score (average of the product of ligand and receptor expression) to define cell type ligand receptor interaction; Identification of significant ligand-receptor interaction between symptomatic and asymptomatic cells by comparing the distributions of cell-cell ligand-receptor interaction scores from symptomatic and asymptomatic cells using Welch’s t-test (Benjamini-Hochberg adjusted *P* < 0.05) and log2 fold change > 0.5
Skelly et al. (2018)		Frequency statistics of ligand-receptor pairs (selecting ligands and receptors expressed at least 20% of cell clusters between cell types)
Wang et al. (2019)		SoptSC: frequency statistics of directed ligand-receptor pairs involving pathways with a probability model based on the cell-cell signaling network
Camp et al. (2017)		Frequency statistics of ligand-receptor pairs between cells (selecting ligands and receptors expressed in each cell)
Cohen et al. (2018)		Analysis of ligand-receptor pairs with ρ > 0.4 between meta-cells as well as prior knowledge to define cell-cell communication
Xiong et al. (2019)		Frequency statistics of highly expressed ligand genes in NASH compared to that in healthy condition between cell types (Fold change > 3) and receptor genes expressed in at least one cluster (normalized UMI > 1.0)
Zhang et al. (2018)	NA	Prior knowledge to define cell-cell communication; Expressed ligands and receptors involving signaling pathway and proteins involving gap junction to study cell-cell communication
Zepp et al. (2017)		Prior knowledge to define cell-cell communication; Expressed ligands and receptors to study cell-cell communication
Duan et al. (2018)		
Li et al. (2017)		Prior knowledge to define cell-cell communication; Expressed ligands and receptors involving signaling pathway to study cell-cell communication
Rajbhandari et al. (2019)		Prior knowledge to define cell-cell communication and the ligand-receptor interacting pair

Validation of inferred cell-cell communicating pairs is the last but most important step. Currently, there are mainly two approaches to validate the inferred pairs, namely histological section analysis of the spatial location of communicating cells and the interacting molecules (ligands and receptors) marked by fluorescence *in situ* hybridization (FISH), immunohistochemistry (IHC) or immunofluorescence (IF). Perturbation experiments (*in vivo* or *in vitro*) using inhibitors or RNA interference (RNAi) are also taken to verify the key ligands and receptors that mediate the cell-cell communication. Concordantly, most inferred communicating cells exhibited observable spatial vicinity on the tissue section such as megakaryocytes-neutrophils in bone marrow, circulating tumor cells (CTC)-neutrophils, extravillous trophoblast (EVT)- decidual natural killer (dNK) in fetal placenta, basophils-macrophages in lung, Oocytes- granulosa cells (GCs), and fetal germ cells (FGCs)-gonadal niche cells in testis, etc. (Table 3).

**Table 3:** Inferred cell-cell communications and validation

Study	Inferred cell-cell communication	Validation of inferred cell-cell communication
Boisset et al. (2018)	Megakaryocytes-neutrophils, Lgr5+ stem cells-Paneth cells, Lgr5+ stem cells-Tac1+ enteroendocrine cell, etc.	Marking the communicating cells by Single-molecule FISH staining on bone marrow and small intestine sections indicated they are significant neighbors
Szczerba et al. (2019)	CTC-neutrophils	Marking the CTC and neutrophils by IF staining indicated they are primarily neighbors; *In vivo* perturbation experiments indicated mice injected with CTC-neutrophil clusters survived for a shorter amount of time compared to those injected with CTCs alone
Martin et al. (2019)	MNPs-T cells, etc.	NA
Kumar et al. (2018)	Cancer cells-CAFs, Cancer cells-macrophages	NA
Vento-Tormo et al. (2018)	EVT-dNK cells	Marking the EVT and dNK cells by IHC staining on decidual serial sections indicated they are primarily neighbors.
Hu et al. (2019)	PCs-RPE cells	NA
Fernandez et al. (2019)	T cells-macrophages	NA
Skelly et al. (2018)	Macrophages-pericytes, Macrophages-fibroblasts	NA
Wang et al. (2019)	HSPC-Monocytes; HSPC-granulocytes, etc.	NA
Camp et al. (2017)	HE cells-macrophages, HE cells-endothelial cells	*In vitro* perturbation experiments by knocking down the key ligand *EDN1* of co-cultured endothelial cells indicated the differentiation of co-cultured HE cells was significantly impaired
Cohen et al. (2018)	Alveolus-Basophils, Basophils-macrophages	Marking the communicating cells by IHC staining of lung sections indicated their spatial proximity to each other; *In vitro* perturbation experiments and *in vivo IL-33* receptor knockout mice experiments indicated basophils regulate alveolar macrophage maturation and immunomodulation functions
Xiong et al. (2019)	HSCs-endothelial cells; HSCs-macrophages; HSCs-T cells, etc.	NA
Zhang et al. (2018)	Oocytes-GCs	Marking the oocytes and GCs specific protein involving gap junctions by IHC staining indicated they are primarily neighbors
Zepp et al. (2017)	Mesenchymal cells-AT2	Spatial distance mapping using Leica indicated the adjacent location of Mesenchymal cells and AT2; *In vitro* perturbation experiments using alveolar organoid indicated the ability of mesenchymal lineages to promote alveolar organoid growth
Duan et al. (2018)	PDGFRb cells-neurons	*In vitro* and *in vivo* perturbation experiments with RNAi indicated PDGFRb cells communicate neurons by secreting chemokine *CCL2* during early infection
Li et al. (2017)	FGCs-gonadal niche cells	Marking the FGCs and gonadal niche cells specific protein involving BMP and Notch signaling by IF staining of testes indicated the communication between them
Rajbhandari et al. (2019)	IL10 immune cells-adipocytes	*In vivo* perturbation experiments with adipocyte-specific *IL10* receptor-deficient mice indicated the communication of *IL10* immune cells-adipocytes in the modulation of the adipose adrenergic response

For the significantly enriched ligand-receptor interactions in cell-cell communication network, perturbation experiments are currently conducted to verify the interaction between communicating cells through inhibiting a key gene or protein that regulates the cell-cell communication (e.g., interacting molecules including ligands or receptors). The cell function for *in vitro* validation or organic function for *in vivo* validation is compared to the observations without perturbation (control) to validate inferred cell-cell communications. The interactions of CTC-neutrophils, mesenchymal cells- alveolar type 2 (AT2) in lung, PDGFRb cells-neurons in brain, and IL10 immune cells-adipocytes (shown in Table 3) are found through this approach.

## Strategy of studying cell-cell communication

**Figure 2:**
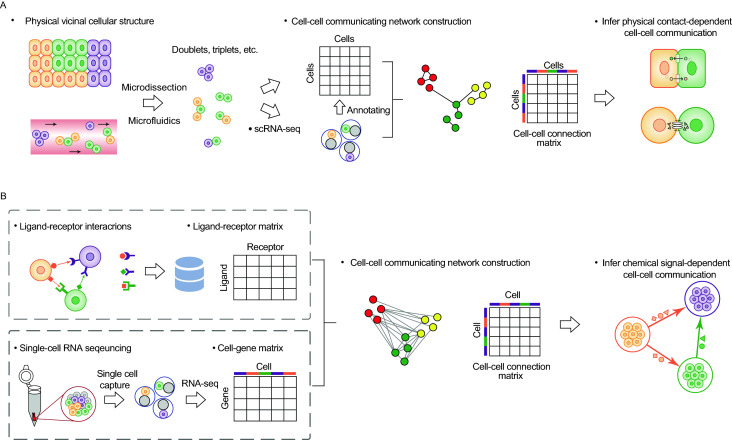
**Two strategies used to investigate cell-cell communications at a single-cell resolution**. (A) Physically vicinal structure-based strategy according to physical contact-dependent communication. Physically vicinal cellular structures (doublets, triplets, etc.) are obtained by microdissection or microfluidics followed by processing scRNA-seq protocols. After annotation of cell types of physically vicinal cellular structures, the cell-cell communicating network is constructed combined with the cell-cell connection matrix for inference of physical contact-dependent cell-cell communication. (B) Ligand-receptor interaction-based strategy according to chemical signal-dependent communication. A ligand-receptor matrix is obtained from known ligand-receptor interactions and a cell-gene matrix is generated from scRNA-seq protocols. The matrices are integrated to construct the cell-cell connection matrix and the cell-cell communicating network using. The chemical signal-dependent cell-cell communication can be further inferred based on the constructed cell-cell communicating network

In order to study cell-cell communication at a single-cell resolution, it is necessary to infer the communicating relationships between the different cell-types and to determine the mechanism of the communicating molecules. After annotation of the cell identities with known cell makers (Zhang et al., 2019) or reference database, cell-cell communication between marked cells can be systematically determined using computational methods based on cell-cell communicating network analysis and further validated with *in vitro* or *in vivo* experiments. Currently, there are two widely adopted strategies to derive cell-cell communications from scRNA-seq data, namely physically vicinal structure-based and ligand-receptor interaction-based strategies (Fig. 2). These two strategies are classified according to the definition of cell-cell communicating modes, e.g., physical contact-dependent or chemical signal-dependent communications).

### Physically vicinal structure-based strategy

In multicellular organisms, cell function are often perturbed when neighboring cells dysfunction or are absent, which indicates the importance of physical contact-dependent communication during biological processes (Stagg and Fletcher 1990). Therefore physically vicinal structure of cells would provide important information on cell-cell communication. Based on this assumption, Boisset et al. developed ProximID, a method to help identify new preferential cellular interactions in the absence of prior knowledge (Boisset et al., 2018). In brief, ProximID applied microdissection to collect small interacting cell structure from mouse bone marrow (cell doublets, triplets, etc.). Cell identities present in the dissected structures were determined using transcriptomic data from scRNA-seq (Fig. 2A). Cell-cell connection matrix was obtained from small physically vicinal structures of cells generated by microdissection. Single cells constitute the network nodes and the spatially physical connection of cells constitutes the network edges.

The permutation test on the cell labels of the physically interacting structure helped to generate a simulated cell-cell communicating network as a background model. Significantly enriched cell-cell communicating pairs were recognized by comparing the number of cell-cell connection in the actual cell-cell communicating network to that in the background model (Table 2). According to their results, enriched communications between *Lgr5*+ stem cells and Paneth cells in small intestinal crypts were found, which was consistent with previously published results. ProximID also identified erythroblastic islands, an important niche in the bone marrow for red blood cellular maturation. In addition, several new cell-cell communications between plasma cells and myeloblasts, megakaryocytes and neutrophils, and *Tac1*+ enteroendocrine cells and *Lgr5*+ stem cell were successfully identified from the constructed cell-cell communicating network based on the statistical method.

For physical contact-dependent cell-cell communication, a common validation practice is to analyze the spatial location of the communicating cells within histological sections of tissue. IHC, IF, or FISH are often used to label the cells of interest with known cell markers. Theoretically, inferred communicating cells are likely to be adjacent in the spatial distribution. For example, Boisset et al. performed single-molecule FISH on bone marrow sections to validate the previously unidentified preferential cell-cell communications *in situ*. By labeling plasma cells and myeloblasts, they visualized and analyzed a large surface area of the bone marrow sections. Combined with statistical analysis, they successfully validated the communication between the plasma cells and myeloblasts by demonstrating that these two types of cells are neighbors of each other within the bone marrow sections with a significantly enriched tendency to physically interact *in situ* (Table 3).

Similarly, Szczerba et al. collected CTCs structures from patients with breast cancer using a microfluidic device and observed that some CTCs structures consisted of the CTCs and white blood cells (WBCs) by staining with known cell-surface markers (Szczerba et al., 2019). By applying scRNA-seq analyzing the transcriptomic data of the associated WBCs, they identified that most white blood cells in the CTCs structures as neutrophils, and determined the physical contact-dependent cell-cell communication between CTCs and neutrophils during cancer dissemination in patients. In addition, Szczerba et al. verified the close interaction between CTCs and neutrophils by comparing the survival time of mice injected with only CTCs and those injected with CTC-neutrophils clusters. Consequently, mice injected with CTC-neutrophil clusters survived for a shorter amount of time compared to those injected with CTCs alone, indicating that the physical contact-dependent communication between neutrophils and CTCs significantly expanded the metastatic potential of CTCs (Table 3).

Drawing upon the power of scRNA-seq techniques, physically vicinal structure-based strategy realizes the study on cell-cell communication at a single-cell resolution by spatially decodes the cell types of small interacting structures from microdissection or microfluidic collection. The resulting communication network closely represents physical connections observed experimentally, which greatly improves the accuracy of computationally inferring cell-cell communication pairs.

However, small interacting structures were mainly collected by manual process based on microdissection devices, leading to the low capturing throughput in a single assay and potential false negatives. Due to its limitation on definition of cell-cell communication, distant cell-cell communications within the tissue microenvironment may be missed by this strategy. In the statistical analysis on communication network, the question remained if the number of cell-cell connection pairs can correlate with the real communication between these two cell types.

### Ligand-receptor interaction-based strategy

For most single-cell studies, the physically spatial locations of cells are lost during the frequently-used scRNA-seq protocol such as 10x Genomics (Zheng et al., 2017). Communication between cells are partly mediated through secreted signaling molecules, such as cytokines and hormones (Sicard 1986; Gartner et al., 2017). Secreted signaling molecules play fundamental roles in chemical signal-dependent cell-cell communications for both physically vicinal cells and distant cells (Braga 2002). With accumulating results about chemical signals for decades, thousands of ligand-receptor interacting pairs have been defined and validated experimentally (Ramilowski et al., 2015), which enables the use of scRNA-seq to construct cell-cell communicating network based on differentially expressed gene levels of ligands and receptors to infer potential cell-cell communications such as CellPhoneDB (Efremova et al., 2020) and SoptSC (Wang et al., 2019). Using available ligand-receptor interactions database (Ramilowski et al., 2015), chemical signal-dependent cell-cell communication can be inferred from the constructed cell-cell communicating network, where the edges represent the interacting intensity integrated from the enriched ligand-receptor pairs between two cells and the nodes denote single cells or cell-types (Fig. 2B).

Camp et al. (2017) sequenced three-dimensional liver bud organoids that were constituted with induced pluripotent stem cell-derived human hepatic endoderm (HE), macrophages, and endothelial cells at a single-cell resolution to identify the communications between these cell populations during liver bud development. By constructing and analyzing the cell-cell communicating networks of receptor-ligand pairings, HE cells demonstrated more extensive crosstalk with macrophages and endothelial cells compared to that with other HE cells (Tables 1–3).

To investigate the chemical signal-dependent cell-cell communications under the condition of non-alcoholic steatohepatitis (NASH), Xiong et al. obtained a total of 33,168 single-cell transcriptomes including 17,788 normal liver cells and 15,380 cells under NASH (Xiong et al., 2019). According to highly expressed ligands and receptors identified in NASH, a liver cells’ ligand-receptor communicating network was constructed to investigate intercellular crosstalk within the liver microenvironment of NASH, which included cholangiocytes, hepatic stellate cells (HSCs), hepatocytes, as well as multiple immune cells. Consequently, they determined that HSCs serve as a hub of intrahepatic signaling through the secretion of HSC-derived stellakines to endothelial cells, macrophages, and T cells during NASH by analyzing the constructed communicating network (Tables 1–3)

Using the same strategy, Martin et al. sequenced 82,417 lamina propria cells from 11 patients with ileal Crohn’s disease (iCD) and characterized a GIMATS module (*IgG* PCs, inflammatory MNPs, and activated T and stromal cells) in a subset of iCD patients, namely *IgG* plasma B cells, inflammatory mononuclear phagocytes (MNPs), and activated T and stromal cells (Martin et al., 2019). For patients enriched or lacking the GIMATS module, a ligand-receptor activity networks was constructed in which network edges referred to the normalized ligand and receptor expression from the source to the target cell type. Computational analysis on the intensity scores of each ligand-receptor pair between each pair-wise cell types determined several ligand-receptor interactions related with receptors on T cells and ligands secreted by MNPs were significantly enriched in GIMATS enriched iCD patients, including *CCL19*-*CCR7*, *CCL2*-*CCR4*, and *IL6*-*IL6R* interacting pairs. To explore cell-cell communication during early maternal–fetal interfaces in humans, Vento et al. collected approximately 70,000 individual cells from first-trimester placentas and annotated them according to known marker genes, such as EVT, dNK cells and dendritic cells (DCs) (Vento-Tormo et al., 2018). By considering the expression levels of ligands and receptors within each cell type, numerous significant ligand–receptor pairs involving immunomodulation, adhesion, and recruitment have been identified between EVT and dNK cells (Tables 1–3).

In addition, several novel chemical signal-dependent cell-cell communications underlying crucial biological processes have been identified (Table 3) using this strategy. That include non-myocytic heart cells with normal cardiac function (Skelly et al., 2018), immune and non-immune cells during lung development (Zepp et al., 2017; Cohen et al., 2018), malignant and non-malignant cells of tumor microenvironment (Kumar et al., 2018), and nervous and immune cells against infection (Duan et al., 2018), etc. (Tables 1–3).

Perturbation experiments are usually conducted to validate the inferred cell-cell communication. It is expected that the function or population of one cell type will be influenced by inhibiting the key ligand of the other communicated cell type. For example, Camp et al. (2017) verified extensive crosstalk between HE cells and endothelial cells by knocking down *EDN1*, the key ligand of endothelial cells. Consequently, they found that the differentiation of co-cultured HE cells was significantly impaired. Beside the *in vitro* perturbation experiments with the co-culture on communicating cells, Rajbhandari et al. applied *in vivo* perturbation experiments to verify the cell-cell communications between *IL10*-producing immune cells and adipocytes (Rajbhandari et al., 2019). Compared to the normal mice, mice with knocked out adipocyte-specific *IL10* receptor were protected against weight gain and observed with increased inguinal brown adipose tissue under high-fat diet, suggesting the import role of *IL10*-producing immune cells and adipocytes’ communication in regulating the thermogenesis and systemic energy balance involving diet-induced obesity (Table 3).

To date, intercellular crosstalk remain poorly understood, including the signals that initiate the communication and how the communication is regulated and maintained. scRNA-seq techniques provide new insights into the mechanisms involved in these known cell-cell communications at a single-cell level (Tables 1–3). As an example, Hu et al. analyzed 2,421 individual cells isolated from human fetal neural retinas (NR) and retinal pigment epithelium (RPE) and analyzed them by scRNA-seq (Hu et al., 2019). The results revealed dynamic expression patterns of the visual cycle and ligand-receptor interaction-related genes such as *PTPRZ1*, *MDK*, and *PTN*. Besides, it is known that bidirectional communication of GCs is required for folliculogenesis. To better understand the crosstalk between oocytes and GCs, Zhang et al. evaluated the transcriptomes of the cells using scRNA-seq and recapitulated the dynamic mechanism of transcriptional regulation between oocytes and GCs during folliculogenesis (Zhang et al., 2018). In addition, it has been reported that the communication between epithelial progenitors and the surrounding mesenchymal cells is able to modulate the ability of the epithelial progenitors to proliferate and differentiate. Combined with scRNA-seq evaluation, Zepp et al. identified Pdgfra as the interacting molecule expressed by mesenchymal cells that mediates the growth and self-renewal of epithelial cells (Zepp et al., 2017).

Ligand-receptor interaction-based strategy takes the gene expression level of known ligands and the corresponding receptors into account. Compared to physically vicinal structure-based strategy, this strategy is not only able to infer the vicinal cell-cell communications, but also the distant cell-cell communications through indirect ligand-receptor interactions.

Whereas, it may be difficult to identify contact-dependent communication via transmembrane proteins or gap junctions (Evans 2015) rather than ligand-receptor interactions. Furthermore, the performance of this strategy heavily depends on reference databases of known ligand-receptor interactions. With notable variations on expression levels of these ligand and receptor genes, inference of cell-cell communications based on ligand-receptor interaction may be restricted. It is still under debate how the edges in the network represent multiple bi-directional interactions between cells.

## Current applications

It is a common to study the biological processes including physiological processes, disease pathogenesis and progression, and pharmacological research of pharmacological treatment and drug resistance by focusing on the key genetic events (copy number variations (Pan et al., 2020), mutations (Zheng et al., 2019, etc.), genes (Zhou 2020), RNAs (miRNAs Xu et al. (2019), lncRNAs (Lin et al., 2019, etc.), proteins Shao et al. (2016), signaling pathways (Liao et al., 2018), or key cells (Mittal et al., 2019), etc. However, more and more researchers in science community have realized the importance of cell-cell communication during biological processes. Increasing findings have indicated that cell-cell communication plays crucial roles in a vast of biological processes including growth, development, disease occurrence and development, etc. in multicellular organisms.

**Figure 3:**
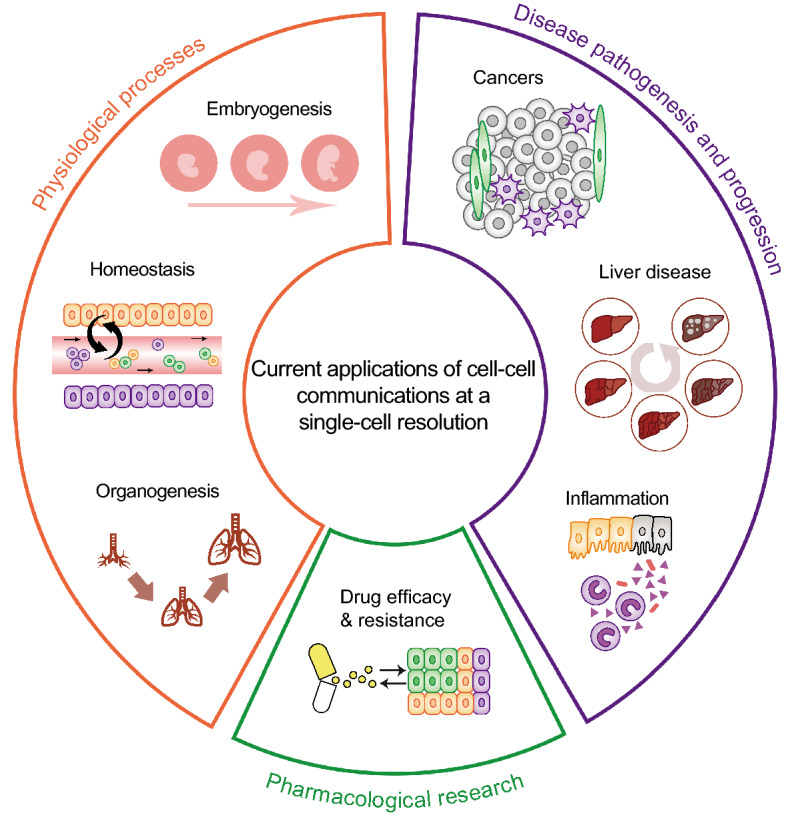
**Current applications of cell-cell communications at a single-cell resolution**. Cell-cell communication studies using scRNA-seq techniques can be applied to elucidate in-depth mechanisms underlying physiological processes (e.g., embryogenesis, homeostasis, and organogenesis), disease pathogenesis and progression (cancers, liver diseases and inflammation), or pharmacological research for efficacy and resistance

Basically, scRNA-seq-based cell-cell communication studies can be applied to reveal the in-depth mechanisms as they can elucidate the signals that initiate the communication and how the communication is regulated and maintained underlying crucial biological processes (Shalek et al., 2014; Burns et al., 2015). Great efforts have been devoted to related fields including physiological processes, such as embryogenesis (Li et al., 2017), homeostasis (Boisset et al., 2018), and organogenesis (Cohen et al., 2018; Scott and Guilliams 2018), as well as disease pathogenesis and progression for cancers (Tirosh et al., 2016; Kumar et al., 2018), liver diseases (Xiong et al., 2019) and inflammation (Duan et al., 2018; Martin et al., 2019), and pharmacological research of pharmacological treatment and drug resistance (Martin et al., 2019) (Fig. 3). For example, Camp et al. revealed the key cell-cell communication that potentially regulates liver development and the key signaling molecules (Camp et al., 2017), while Zepp et al. sequenced lung mesenchymal cells at a single-cell resolution and identified epithelial-mesenchymal communications critical for lung homeostasis and regeneration (Zepp et al., 2017). In addition, Xiong et al. applied scRNA-seq to liver cells and determined the hepatic stellate cells as the core origin of secreted stellakines during the development of NASH (Xiong et al., 2019). For pharmacological research, Martin et al. applied single-cell technologies to iCD lesions and concluded that the GIMATS module was driven by MNPs and observed a high correlation exists between the GIMATS module and anti-TNF treatment resistance, suggesting that these may serve as novel biomarkers of treatment response and may be exploited for tailored therapeutic opportunities (Martin et al., 2019). Obviously, a better understanding of the cell-cell communications during disease pathogenesis and progression may provide insights into novel therapeutic strategies and targets in pharmacological research (i.e., key cell types and key ligand-receptor interactions), despite the limited number of studies available with respect to cell-cell communication at a single-cell resolution.

## Perspectives

In multicellular organisms, cell-cell communication play crucial roles in numerous biological processes including growth, development, disease occurrence and development, etc. Traditionally, exploring cell-cell communications majorly relies on a large number of experiments such as histological section analysis of the cellular spatial location and *in vitro* or *in vivo* experiments such as co-culture of cells and genetic knockout or knockdown of model organisms. Taking the early characterized cell-cell communication of erythroblastic island as an example (Bessis 1958), Marcel Bessis originally discovered the erythroblastic island about 60 years ago, which depends on his careful analysis of the transmission electron micrographs of bone marrow sections. Based on the substantial experiments, the mechanism and function underlying erythroblastic island have been fully elucidated gradually (Manwani and Bieker 2008). Besides, some microfluidics-based cell co-culture technology also emerges by detecting ligand-receptor interactions or cell migration to discover the dynamic cell-cell communication (Rothbauer et al., 2018). Nevertheless, for known cell-cell communication, how communicating signals mediate cell-cell communications are poorly understood. Besides, the questions about what cell-cell communication initiates certain diseases and how diseases regulate cell-cell communications remain to be answered.

With the advancement of single-cell techniques like the widely-used scRNA-seq, more and more attention has been re-attracted in science community on the investigation of cell-cell communication. Compared to traditional approaches, scRNA-seq can classify single cells into different cell types or subtypes resolving the cellular heterogeneity. On the one hand, scRNA-seq enables the systematic characterization of molecular mechanism underlying the known cell-cell communications that are poorly understood. On the other hand, scRNA-seq enables the more comprehensive and specific investigation of unknown cell-cell communications at a single-cell resolution. However, the accuracy of inferring cell-cell communications with single-cell transcriptomic data is heavily dependent on the computational analysis. Future development of computational methods are required to improve the inference of cell-cell communications. Also, the inferred cell-cell communications and communicating molecules need to be further verified.

Great progresses have been made on detection and analytic techniques for single-cell measurements of copy number variation, protein abundance and metabolic level, etc. (Vitak et al., 2017; Swaminathan et al., 2018; Collins and Aebersold 2018; Zhang and Vertes 2018). Good examples are single-cell proteomic and metabolic techniques including parallel sequencing (Swaminathan et al., 2018; Collins and Aebersold 2018), single-cell mass cytometry (CyTOF) (Bandura et al., 2009), and single cell proteomics by mass spectrometry (SCoPE-MS) (Budnik et al., 2018). Single-cell genomics and transcriptomics reflect the cellular genealogy and can track cells as they evolve and change through mutations (Marx 2019). Single-cell proteomics can be used to classify cells into known cell types or to identify unknown or rare cell types/subtypes according to cell markers, for the inference of physical contact-dependent cell-cell communications by annotating spatially vicinal identity of single cells. Given the inference of chemical signal-dependent cell-cell communications, single-cell proteomics can capture the direct abundance of signaling proteins, while single-cell metabonomics can examine the content of signaling metabolites such as hormone (Pfaff and Baum 2018), neurotransmitter (Sugiyama et al., 2019).

However, high-throughput single-cell methods have not yet arrived in proteomics and metabonomics because of lots of factors such as dyes falling off, low abundance in single cell, infeasibility of amplification like DNA or RNA, challenges in sample and buffer preparation or high cost, etc. (Zhang and Vertes 2018; Marx 2019). Additionally, for some cell-cell communications via hormone, neurotransmitters, the challenges might exist in the annotation of cells and quantification of receptors when using single-cell metabonomics technique.

In consideration of the developed high-throughput scRNA-seq techniques (Cao et al., 2017), it has been a common practice to use mRNA concentrations as proxies for the concentrations and activities of the corresponding proteins, assuming that gene expression levels are the main determinant of protein abundances (Vogel and Marcotte 2012). Compared to genome and proteome, transcriptome analysis provides knowledge of the molecular linkages between genetic information and the proteome, leading to a comprehensive understanding of biological processes including the cell-cell communications (Song et al., 2019; Shao et al., 2019). Undoubtedly, there are some limitations of scRNA-seq for the investigation of cell-cell communications. First, scRNA-seq offers an indirect reflection of protein levels, not a direct measurement. Besides, for cell-cell communications via small signaling molecules such as dopamine and histamine, scRNA-seq can hardly infer this kind of cell-cell communications. Even so, increasing studies have focused on this technique to infer cell-cell communications and the fact holds that scRNA-seq is proved to be an efficient approach to systematically infer and study cell-cell communications combined with computational analysis in recent years.

**Figure 4:**
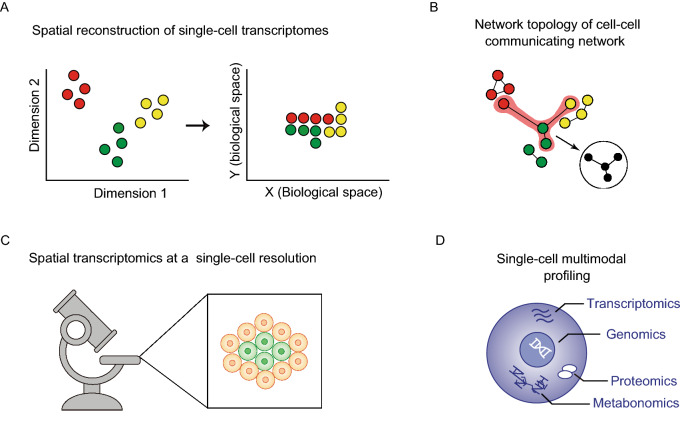
**Challenges and opportunities of investigating cell-cell communication at a single-cell resolution**. (A) Spatial reconstruction of single-cell transcriptomes from single-cell transcriptomic data without spatial location will shed light on the integration of physical contact-dependent and chemical signal-dependent cell-cell communications. (B) Incorporation of network topology and features will help infer cell-cell communications. (C) Recent advances in spatial transcriptomics at a single-cell resolution will facilitate the identification of single-cell intercellular communications *in situ*. (D) Establishing the comprehensive molecular view of the cell by multimodal profiling in the future will definitely benefit the inference of cell-cell communicating modes

In general, cells communicate and interact with each other intricately within the tissue microenvironment, wherein the chemical signal communications may also occur between the physically vicinal cells (Stagg and Fletcher 1990). It is possible for physical vicinal cells to communicate through chemical signals. If incorporated with the information of physical cell-cell connection, it will be more reliable to infer the cell-cell communications combined with ligand-receptor interactions at a single-cell resolution. However, the fact is that it is difficult for most studies currently to integrate two kinds of cell-cell communications within a single assay for the widely-used scRNA-seq protocols lacking of the spatial location of cells. Therefore, the challenge becomes the spatial reconstruction of single-cell transcriptomes from single-cell transcriptomic data, which will shed light on the integration of physical contact-dependent and chemical signal-dependent cell-cell communications (Fig. 4A). Fortunately, spatial reconstruction of single-cell transcriptomes has attracted much attention recently and future improvements in this area may help address the limitations (Satija et al., 2015; Halpern et al., 2017; Nitzan et al., 2019).

The concept of networks is important in many fields, including social sciences, physics, artificial intelligence, ecosystems, and systems biology. Analysis on network topology and features may help scientists predict system behavior (Albert et al., 2000; Barabasi and Oltvai 2004; Xue et al., 2020). Nevertheless, there are limited methods based on network analysis for predicting both physical contact-dependent and chemical signal-dependent cell-cell communications from constructed cell-cell communicating network (Fig. 4B). More efforts should be directed to the development of network analysis methods for cell-cell communicating networks.

With the great advances in spatial transcriptomics-related techniques over the recent years (Wang et al. 2018; Eng et al., 2019; Rodriques et al., 2019), it is likely that future single-cell spatial position and single-cell transcriptome analysis will be able to simultaneously measure both the single cells and the sum of mRNA molecules with a high throughput, especially for specimens originating from solid tissues (Fig. 4C). These future advancements will provide hope for spatially reconstituted transcriptomes *in situ* at single-cell levels and will serve as new inspiration for the advancement of both physical contact-dependent and chemical signal-dependent cell-cell communication studies.

More recently, techniques on multimodal single-cell measurements has drawn increasing attention (Fig. 4D), aiming at the simultaneous profiling on multiple types of molecule within a single cell (Stuart and Satija 2019; Zhu et al., 2020). For example, CITE-seq (Stoeckius et al., 2017) and REAP-seq (Peterson et al., 2017) have realized the simultaneous measurements of whole transcriptome of mRNA and proteins, while sci-CAR have enabled the measurements of whole transcriptome of mRNA and chromatin accessibility simultaneously (Cao et al., 2018). The establishment of a comprehensive molecular view of the cell by multimodal profiling will definitely improve the classification of cell identities and inference of multiple cell-cell communicating modes via biomacromolecule and small signaling molecules.

With the increasing researches on the investigation of cell-cell communications in the future, we expect that more cell-cell communications related with physiological processes, disease pathogenesis and progression, and pharmacological research of pharmacological treatment and drug resistance will be discovered and verified, providing new insights into the old biological and biomedical questions such as mechanism elucidation, identification of biomarkers and drug targets, and drug resistance, etc. As the relevance of cell-cell communications involved in the initiation and development of physiological process and disease becomes better defined, novel or improved therapeutic strategies for pharmacological treatments targeting driving cell types and communicating molecules will become apparent.

## Abbreviations

AT2: alveolar type 2
CAFs: cancer-associated fibroblasts
CTCs: circulating tumor cells
DCs: dendritic cells
dNK: decidual natural killer
EVT: extravillous trophoblast
FGCs: fetal germ cells
FISH: fluorescence *in situ* hybridization
GCs: granulosa cells
HE: hepatic endoderm
iCD: ileal Crohn’s disease
IF: immunofluorescence
IHC: immunohistochemistry
iPS: induced pluripotent stem
MNPs: mononuclear phagocytes
NASH: non-alcoholic steatohepatitis
NR: neural retina
PCs: photoreceptor cells
RNAi: RNA interference
RPE: retinal pigment epithelium
scRNA-seq: single-cell RNA-sequencing
TAMs: tumor-associated macrophages
WBCs: white blood cells
